# In‐field evaluation of Xpert® HCV viral load Fingerstick assay in people who inject drugs in Tanzania

**DOI:** 10.1111/liv.14315

**Published:** 2019-12-15

**Authors:** Zameer Mohamed, Jessie Mbwambo, John Rwegasha, Nicodem Mgina, Basra Doulla, Promise Mwakale, Edouard Tuaillon, Stephane Chevaliez, Yusuke Shimakawa, Simon D. Taylor‐Robinson, Mark R. Thursz, Ashley S. Brown, Maud Lemoine

**Affiliations:** ^1^ Department of Hepatology Imperial College London St Mary's Hospital London UK; ^2^ Department of Psychiatry Muhimbili National Hospital Muhimbili University of Health and Allied Sciences Dar es Salaam Tanzania; ^3^ Department of Gastroenterology Muhimbili National Hospital Dar es Salaam Tanzania; ^4^ Tanzania Central Tuberculosis Reference Laboratory Muhimbili National Hospital Dar es Salaam Tanzania; ^5^ Department of Bioethics Muhimbili University of Health and Allied Sciences Dar es Salaam Tanzania; ^6^ INSERM U 1058 Université Montpellier 1 Montpellier France; ^7^ Département de Bactériologie‐Virologie CHU Montpellier Montpellier France; ^8^ Department of Virology French National Reference Center for Viral Hepatitis B, C and Delta Hopital Henri Mondor Université Paris‐Est Créteil France; ^9^ Unité d'Épidémiologie des Maladies Émergentes Institut Pasteur Paris France

**Keywords:** hepatitis C viurs (HCV) diagnosis, people who inject drugs (PWID), point‐of‐care (POC), sub‐Saharan Africa, Xpert

## Abstract

**Background:**

Although novel hepatitis C virus (HCV) RNA point‐of‐care technology has the potential to enhance the diagnosis in resource‐limited settings, very little real‐world validation of their utility exists. We evaluate the performance of HCV RNA quantification using the Xpert^®^ HCV viral load Fingerstick assay (Xpert^®^ HCV VL Fingerstick assay) as compared to the World Health Organisation pre‐qualified plasma Xpert^®^ HCV VL assay among people who inject drugs (PWID) attending an opioid agonist therapy (OAT) clinic in Dar‐es‐Salaam, Tanzania.

**Methods:**

Between December 2018 and February 2019, consecutive HCV seropositive PWID attending the OAT clinic provided paired venous and Fingerstick samples for HCV RNA quantification. These were processed onsite using the GeneXpert^®^ platform located at the Central tuberculosis reference laboratory.

**Results:**

A total of 208 out of 220 anti‐HCV‐positive participants recruited (94.5%) had a valid Xpert^®^ HCV VL result available; 126 (61%; 95% CI 53.8‐67.0) had detectable and quantifiable HCV RNA. About 188 (85%) participants had paired plasma and Fingerstick whole blood samples; the sensitivity and specificity for the quantification of HCV RNA levels were 99.1% and 98.7% respectively. There was an excellent correlation (*R* ^2^ = .95) and concordance (mean difference 0.13 IU/mL, (95% CI −0.9 to 0.16 IU/mL) in HCV RNA levels between plasma samples and Fingerstick samples.

**Conclusion:**

This study found excellent performance of the Xpert^®^ HCV VL Fingerstick assay for HCV RNA detection and quantification in an African‐field setting. Its clinical utility represents an important watershed in overcoming existing challenges to HCV diagnosis, which should play a crucial role in HCV elimination in Africa.

**Key points**

- Availability of tests to confirm active hepatitis C virus (HCV) infection remain a significant barrier to scaling‐up care in resource‐limited settings.
- We confirm excellent performance of the Xpert^®^ HCV viral load Fingerstick point‐of‐care assay among people who inject drugs in Dar‐es‐Salaam, Tanzania.
- These encouraging results provide a credible solution improve diagnosis and contribute towards efforts to HCV elimination in this vulnerable population.

## 1 BACKGROUND

The advent of highly efficacious direct‐acting antivirals (DAAs) has provided a springboard to raise the awareness of hepatitis C virus (HCV) as a global health issue and acted as a catalyst to the development of the 2030 World Health Organisation (WHO) viral hepatitis elimination targets.^1^ However, currently the majority of infected individuals (80%) remain undiagnosed especially in low‐and middle‐income countries (LMICs) and only a minority (2% annually) are accessing treatment.^2^, ^3^ As a result, only a handful of countries are currently on track to achieve the ambitious elimination objectives. Thus, a great deal of emphasis has been placed on scaling‐up simplified strategies for HCV screening and linkage‐to‐care.^4^

As LMICs represent 80% of the global HCV epidemic, it is clear that an increased focus is required to address the challenges of case‐finding and treatment in these commonly resource‐limited settings.^5^ Recent times have seen the WHO prequalification of DAAs to ensure the quality control, and fierce competition from generic manufacturers has resulted in a sharp reduction in cost of treatment. Ironically, in many resource‐limited settings the cost of diagnosis exceeds the cost of treatment.^6^ As HCV RNA confirmation is a pre‐requisite for commencing treatment, it is not surprising that access to simple HCV diagnostics in such challenging settings has been underlined as a key priority in order to achieve HCV elimination.

However, the resounding limited access to HCV RNA nucleic acid testing (NAT) in LMICs was emphasised by a recent WHO survey, which reported that 40% of LMICs do not have access to HCV NAT.^7^ One of the novel solutions to mitigate the dependence on skilled laboratories and personnel is the emergence of simple point‐of‐care tests for HCV RNA. Currently, two CE‐in vitro diagnostic point‐of‐care platforms (Genedrive^®^) and Xpert^®^ (Cepheid) for HCV RNA NAT in plasma exist, with one (Xpert^®^) receiving WHO prequalification in 2017.^8^, ^9^

However, with the existing point‐of‐care tests there still remains the issue of plasma separation. Whole blood samples of 50‐70 µL have been demonstrated as an alternative method to decentralise and simplify sample collection using dried blood spots (DBS).^10^ This is particularly beneficial in rural settings and in people who inject drugs (PWID), where venous access is challenging. In addition, the ease of obtaining a sample can allow task‐shifting to a diverse range of healthcare and non‐clinical staff.^11^, ^12^ DBS have also been validated for HCV RNA quantification on the new Hologic Aptima assay as well as HCV core antigen quantification on the Abbott Architect platform, reporting sensitivities of 96.4% and 76.1% respectively.^13^, ^14^ Furthermore, a Fingerstick assay using 100 µL has recently been validated for the Xpert^®^ system (Xpert^®^ HCV viral load [VL] Fingerstick assay), which has a lower limit of detection of 40 IU/mL and lower limit of quantification of 100 IU/mL.^15^, ^16^, ^17^ When compared with the Genedrive^®^ device, not only does the Xpert^®^ HCV VL Fingerstick assay have a superior threshold for HCV RNA detection and avoid the need for any sample prepreparation, it also has a faster turn‐around time (60 minutes vs 90 minutes) and is half the price. All of which make it quite an attractive proposition to improve the access to HCV RNA testing in resource‐limited settings.

Although HCV RNA point‐of‐care assays have been designed for LMICs, a lack of in‐field validation data in such environments exists and addressing this has recently been highlighted as crucial.^18^ For example, the urgent need to scale‐up resources to address the risk of blood‐borne viruses (BBVs) among PWID engaged in harm reduction in Tanzania has previously been highlighted, including the pressing need for locally available HCV RNA confirmation as part of the cascade of care.^19^, ^20^ In addition, a lack of access for temperature regulated sample transport and storage, particularly in a tropical climate, provide further justification for the need to confirm the performance of the Xpert^®^ HCV VL Fingerstick assay in this resource‐limited African setting. Thus, this study population, attending a opioid agonist therapy (OAT) clinic in Dar‐es‐Salaam, Tanzania, was used to assess the performance of onsite HCV RNA quantification with Xpert^®^ system by comparing the use of finger‐stick capillary blood sample (Xpert^®^ HCV VL Fingerstick assay) to the use of plasma sample (Xpert^®^ HCV VL) as a WHO prequalified reference.

## 2 MATERIALS AND METHODS

### 2.1 Study population

The Muhimbili National Hospital (MNH), Dar‐es‐Salaam, Tanzania is the national referral hospital for the country. Situated onsite, the OAT clinic is the product of a Tanzanian government and President's Emergency Plan For AIDS Relief supported initiative that has been operational since 2011.^19^ In addition to offering daily OAT to all clients, routine serology screening for BBVs (HIV, hepatitis B and HCV) is offered on enrolment to the clinic. Currently, linkage‐to‐care is limited to HIV and TB, while HBV‐negative patients are offered vaccination. Clients can also seek aid from a wide range of services centralised at the clinic including; psychiatry, social welfare, nursing (for eg wound care) and can also enrol in a harm reduction mentorship program to become a community outreach worker. There is also an evolving link with the Department of Gastroenterology and Hepatology at MNH, which has been nurtured as part of this collaboration. Currently, access to generic DAA therapy is available on a private basis only, which is not readily accessible to HCV‐infected PWID. In order to assess the performance of HCV RNA quantification, all clients with a known positive result for anti‐HCV antibody attending the clinic for OAT, irrespective of concomitant illicit drug use, were invited to participate in the study between December 2018 and February 2019.

### 2.2 Ethical considerations

This study received clearance from the respective institutional review board at MNH (ref: MNH/IRB/1/2018/178). All participants provided written informed consent in line with good clinical practice.

### 2.3 Clinical assessment and sample collection

All recruited individuals completed a comprehensive demographic and risk factor‐based questionnaire, which was administered by a trained fieldworker. All participants then provided paired blood samples. Venous samples were obtained to fill a 4 mL ethylenediamine tetraacetate (EDTA) bottle, while a 100 µL finger‐prick sample was collected in an EDTA‐coated capillary (Minivette^®^; Sarstedt) (http://www.sarstedt.com) and applied directly into the Xpert^®^ HCV VL Fingerstick assay cartridge in line with the manufacturer's specifications (Cepheid).^15^ All samples were then delivered to the onsite Central TB Reference Laboratory at MNH for processing on the day of sample collection.

### 2.4 Laboratory processing and virological assessment

Laboratory staff prioritised processing Xpert^®^ HCV VL Fingerstick assay samples on the same day as receipt on the GeneXpert^®^ II platform in accordance with the manufacturer's guidance, to provide a quantitative HCV RNA result within 60 minutes. All venous samples were kept at 4°C for a maximum of 72 hours prior to plasma separation, 1mL was applied to the Xpert^®^ HCV VL cartridge before being processed for HCV RNA quantification on the GeneXpert^®^ II platform in accordance with the manufacturer's guidance. All residual plasma was stored at −80°C. In the event that either plasma or finger sample failed, where possible a repeat paired sample was collected and run on the Xpert^®^ platform.

### 2.5 HCV genotyping

Where available historic HCV genotype was used as previously reported.^13^ Previously described methods were used to perform sequencing of the HCV genome NS5B region and subsequent phylogenetic analysis.^21^

### 2.6 Sample size calculation

We aimed to demonstrate that a lower boundary of a 95% confidence interval of the diagnostic sensitivity of Xpert^®^ HCV VL Fingerstick assay is at least higher than 90% with a power of 80%. Assuming that the prevalence of chronic HCV infection is 60%‐80% in anti‐HCV antibody‐positive patients, and the true sensitivity of Xpert^®^ HCV VL Fingerstick assay is 97%, we needed to recruit at least 184 anti‐HCV‐positive patients.

### 2.7 Statistical analysis

Characteristics of the study participants were presented by mean, standard deviation, while non‐parametric continuous variables were presented by median and interquartile range (IQR) and compared using the Mann‐Whitney *U* test. Categorical variables were presented by percentage. Accuracy of Xpert^®^ HCV VL Fingerstick assay to correctly identify HCV RNA‐positive patients was presented by sensitivity and specificity. Correlation and agreement of HCV RNA quantification between Xpert^®^ HCV RNA plasma and Xpert^®^ HCV VL Fingerstick assay were presented by Pearson's correlation coefficient and Bland Altmann analysis respectively. All statistical analyses were completed using Microsoft Excel 2010 and SPSS statistical software version 24.0.

## 3 RESULTS

### 3.1 Participant characteristics

During the study period, 220 anti‐HCV positive patients agreed to participate. The cohort was overwhelmingly male (98%) and had a median age of 41 years (IQR: 37‐45). All had a past history of injecting drug use, on average clients started to inject drugs at the age of 23 years with 38% admitted to having ever‐shared needles in the past. The median length of time enrolled in the OAT at the time of recruitment was 5 years. The study population characteristics are presented in Table 1.

**Table 1:** Anti‐HCV‐positive participant characteristics (n = 220)

Variable	Result (N = 220)
Median age, y (IQR)	41 (37‐45)
Male sex (n, %)	216 (98)
HIV co‐infection (n, %)	67/185 (36)
Median age starting injecting drugs (IQR)	23 (20‐27)
Needle sharing (n, %)	69/182 (38)
Median years on opioid substitution therapy (IQR)	7 (5‐8)
Positive HCV RNA (n, %)	126/208 (61)
Median HCV RNA viral load (range) (log IU/mL)	6.1 (1.4‐7.5)
Genotype (n, %)	
1a	35/53 (66)
4a	18/53 (34)

Abbreviations: HCV, hepatitis C virus; IQR, interquartile range.

### 3.2 HCV RNA assessment

**Figure 1:**
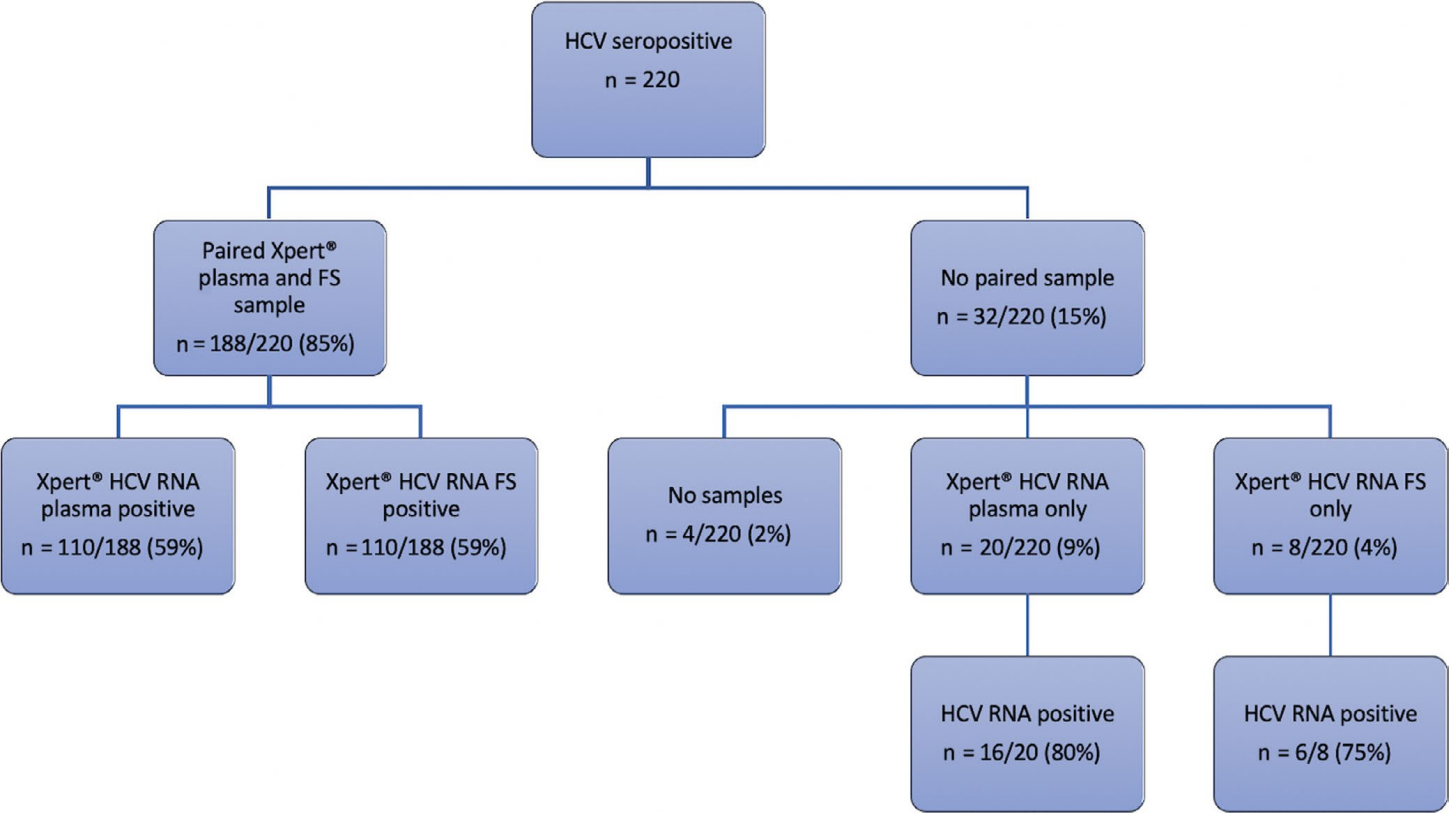
Summary of hepatitis C virus (HCV) nucleic acid testing pathway for anti‐HCV antibody positive patients

About 208 of the 220 (95%) of clients enrolled in the study had a valid plasma Xpert^®^ HCV VL result. Of the 12 (5%) without a valid reference HCV RNA result, a venous sample could not be obtained in 8 (4%) clients owing to difficult venous access, three (1%) clients failed owing to inadequate sample volume and one failed owing to a power cut during sample processing. One hundred and twenty‐six clients had quantifiable HCV RNA, resulting in a prevalence of chronic HCV infection of 61% (95% CI 54‐67). All viraemic patients had quantifiable HCV RNA, with a median HCV RNA level of 6.1 log IU/mL (range; 1.4‐7.5). Genotype was available for 53 (42%), with genotype 1a identified in 35 of 53 cases (66%) and genotype 4a in 18 of 53 (34%) cases respectively (Figure 1).

### 3.3 Repeat sampling and error rates

**Figure 2:**
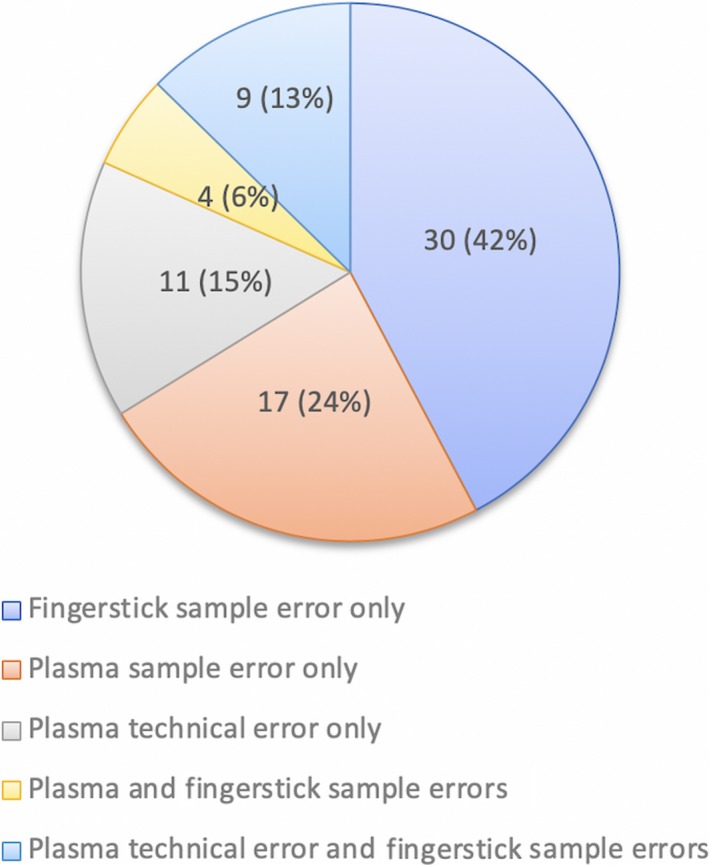
Breakdown of testing errors for both Xpert^®^ HCV RNA plasma and Fingerstick assay (n = 71). HCV, hepatitis C virus; VL, viral load

Overall 71 (32%) of patients required repeat testing owing to test errors (30/71 (42%) Xpert^®^ HCV VL Fingerstick assay only, 28/71 (40%) plasma Xpert^®^ HCV VL only and 13/71 (18%) both assays). In the case of plasma Xpert^®^ HCV VL, 21 of 41 (51%) patients were related to sample volume errors and 20 of 41 (49%) were related to technical failure, which in most cases was owing to instrument interruption after a power shortage. In comparison, all 43 (100%) of the Xpert^®^ HCV VL Fingerstick assay errors were related to inadequate sample volume. Consequently, 32 of 220 (14.5%) patients did not have a paired sample; 4 of 220 (2%) had no sample, 20 of 220 (9%) had a plasma sample only and 8 of 220 (4%) had an Fingerstick sample only (Figures 1 and 2).

Date of plasma Xpert^®^ HCV VL and Xpert^®^ HCV VL Fingerstick testing was available for 213 of 220 (97%) and 206 of 220 (94%). For plasma Xpert^®^ HCV VL and Xpert^®^ HCV VL Fingerstick, errors occurred in 13 of 65 (19%) and 7 of 65 (11%) tests in December, 28 of 130 (22%); and 28 of 126 (22%) tests in January; and none of the 14 (0%) and 4 of 15 (27%) in February respectively. Date of testing was not available for the additional four error samples that occurred in Xpert^®^ HCV VL Fingerstick testing.

### 3.4 Performance of Xpert^®^ HCV RNA Fingerstick assay

**Figure 3:**
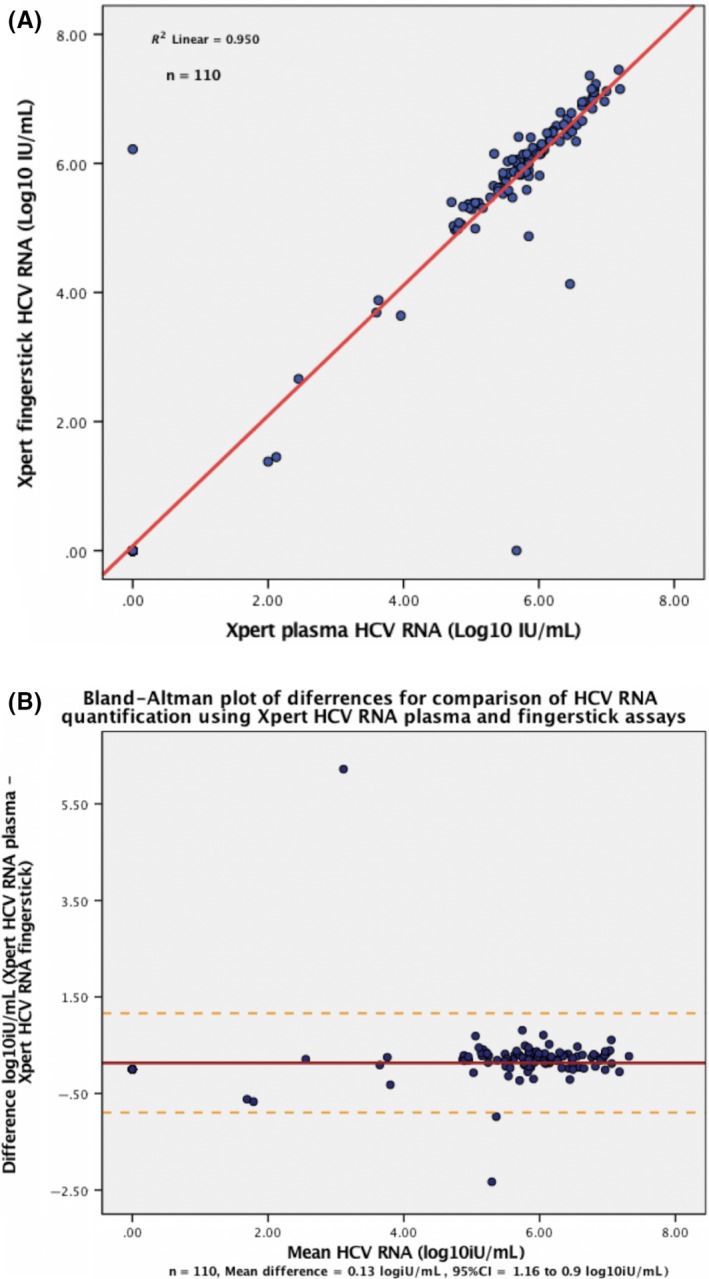
A, Regression plot comparing HCV RNA for plasma Xpert^®^ HCV VL and Xpert^®^ HCV VL Fingerstick assays. B, Bland‐Altman plot of difference comparing HCV RNA for plasma Xpert^®^ HCV VL and Xpert^®^ HCV VL Fingerstick assays. HCV, hepatitis C virus; VL, viral load

About 188 (85%) of those enrolled had paired plasma and finger‐stick samples analysed. Viraemia was considered if HCV RNA was both detected and quantified in using each respective test. Overall the Xpert^®^ HCV VL Fingerstick assay performed excellently, with a sensitivity of 99.1% (95% CI 95.0‐100) and specificity of 98.7% (95% CI 93.1‐100) (Table 2). Two discrepant results were identified, one where the HCV RNA was detectable in plasma (6.2 Log IU/mL) and undetectable in the Fingerstick sample, while one which was only detectable in Fingerstick whole blood (5.7 Log IU/mL) and undetectable in plasma. In addition, the correlation between Xpert^®^ HCV VL plasma and Xpert^®^ HCV VL Fingerstick quantification was near perfect (*R* ^2^ = .95) (*P* < .001). There was a strong level of agreement of HCV RNA quantification (mean difference 0.13 Log IU/mL; 95% CI: −0.9 to 1.2 Log IU/mL) (Figure 3A,B).

**Table 2:** Performance of Xpert^®^ HCV RNA finger‐stick assay as compared to Xpert^®^ HCV RNA plasma assay (n = 188)

n = 188	Reference test: Xpert^®^ HCV VL plasma assay (cut‐off 4 IU/mL)		
Index test: Xpert^®^ HCV VL Fingerstick assay (cut‐off 40 IU/mL)	HCV RNA quantifiable	HCV RNA negative	Total
HCV RNA quantifiable	109	1	110
HCV RNA negative	1	77	78
Total	110	78	188

Note

Xpert^®^ HCV viral load (VL) Fingerstick assay; sensitivity 109/110 (99.1%), specificity 77/78 (98.7%).

Abbreviation: HCV, hepatitis C virus.

## 4 DISCUSSION

The challenges of accessing NAT for HCV RNA confirmation has been raised as a key barrier to improving diagnosis in resource‐limited settings in sub‐Saharan Africa and more specifically in the PWID population in Tanzania.^13^, ^19^, ^22^, ^23^ In this study we report the excellent performance of the Xpert^®^ HCV VL Fingerstick assay to confirm viraemic HCV infection. Indeed, our results are consistent with the findings from Lamoury and colleagues, who reported a sensitivity of 98.3% and a specificity of 100% of the Xpert^®^ HCV VL Fingerstick assay to detect HCV RNA.^16^ In our study, two discrepant results were reported; unfortunately, neither of the patients could be re‐traced for repeat testing during the study window. However, it is important to note that the Xpert^®^ HCV VL Fingerstick assay cartridges provided were the first generation research use only versions, which is likely to have a lower quality control in comparison to the recent fully CE marked commercially available assay.

Existing literature evaluating the performance of the Xpert^®^ HCV VL assay in Cambodia has provided compelling evidence to support its use in such resource‐limited settings.^24^ However, this study was conducted using plasma, which still requires a laboratory set up for plasma separation. Our study adds to the limited literature addressing the use of HCV NAT point‐of‐care tests in sub‐Saharan Africa, raising the potential for Xpert^®^ HCV VL Fingerstick assay to provide a further simplified solution to existing issues related to HCV diagnosis. In addition, we add our findings to the limited data supporting the use of the Xpert^®^ platform in HCV genotype 4.^25^, ^26^

In recent times, the challenge for HCV point‐of‐care diagnostic assay development has been to match conventional laboratory‐based NAT lower‐limits of detection, which are as low as 15 IU/mL. The clinical relevance of relaxing this threshold has recently been tested in a global dataset, which identified a HCV RNA cut‐off of 1316 IU/mL would identify 97% of cases.^27^ In addition, a study evaluating VL in DAA treatment relapse suggested that 97% of cases had a VL of greater than 10 000 IU/mL.^28^ The European Association for the Study of Liver has also recently endorsed a HCV NAT diagnostic cut‐off of 1000 IU/mL for LMICs in order to increase HCV RNA point‐of‐care uptake.^29^ Despite these recommendations, very limited real‐world validation of HCV RNA point‐of‐care exists in LIMCs and none in Africa have been published so far. The WHO has pre‐qualified the Xpert^®^ HCV VL assay,^8^ which not only ensures quality control but also is often a prerequisite for donor funded programmes.^6^ However, despite having a lower limit of detection significantly below recommendations (40 IU/mL) from a whole blood sample, the Xpert^®^ HCV VL Fingerstick assay is yet to receive WHO pre‐qualification.

There are a number of advantages specific to the GeneXpert^®^ platform that makes it an attractive option to improving the diagnosis of communicable infections across sub‐Saharan Africa. Firstly, it is already a well‐established technology used as the first‐line diagnostic test for Tuberculosis (TB) in sub‐Saharan Africa. In response to the WHO 2010 recommendations for TB diagnosis,^30^ an exponential rise in Xpert^®^ modules to 21 549 was recorded between 2010 and 2015 in 122 ‘high‐burden developing countries’ (HBDC).^31^ Furthermore, recent data from Nigeria confirm that there are over 1500 modules distributed across the nation.^32^ Thus, it is not only a familiar technology but also widely disseminated across the continent. This is particularly key in resource‐limited settings as in most cases it would avoid the potentially prohibitive USD$17 000 instrument cost.^33^ Secondly, Cepheid has agreed to honour a uniform assay cost of USD$14.90 for HBDC, which may allow government programmes to pool finances and negotiate the best rate possible when purchasing cartridges. Thirdly, the platform can be used simultaneously to test for other endemic viral infections. For example, the WHO have prequalified the Xpert^®^ HIV VL assay, which has a lower limit of detection of 38 IU/mL. Its performance and utility have also been tested in a rural community‐based project in Botswana, reporting a sensitivity of 98.6%.^34^ More recently, Cepheid have launched a hepatitis B VL assay, which may contribute a much‐needed tool to aid treatment decision‐making and monitoring in resource‐limited settings. Finally, the multi‐modular aspect to the GeneXpert^®^ instrument may encourage synergism of human resources between various national disease management strategies. In fact, this project is an example of collaboration with the Tanzanian Central TB reference laboratory who provided access to both the instruments and staff. Additionally, Bwana and colleagues have also demonstrated similar ease of integration of the Xpert^®^ HIV VL assay into TB serves in Kenya.^35^ These combined factors form a compelling rationale for integrating viral hepatitis testing within the existing expansive Xpert^®^‐based TB services across sub‐Saharan Africa. The prospective success of such a hybridised model could provide a paradigm to shape future screening efforts and influence treatment coverage for viral hepatitis in resource‐limited settings.

However, in our study one‐third of patients required re‐testing owing to test errors, which were attributed to both inadequate sample volume and also loss of electrical supply while samples were being analysed. The rate of error appears to be fair greater than that has previous been reported in the literature (1%‐3%).^16^, ^17^ In this study, the majority of errors were experienced with the Xpert^®^ HCV VL Fingerstick assay (43/71, 61%). One difference in our methodology is that samples were transported to the virology laboratory (approximately 10‐minute walking distance). It is therefore conceivable that the act of transportation, in addition to exposure to high temperatures and humidity, may have impacted on the sample viability. An added problem experienced was with the practicality of the Fingerstick sample collection device, which relied on capillary action. Air interruption during sample collection often resulted in inadequate sample volume being obtained and assay failure. Despite re‐training sample collection and processing, recurrent sample error remained an issue throughout the study. It would therefore be important for similar studies to be undertaken in similar resource‐limited settings to corroborate the challenges experienced in our setting and identify other issues, which could minimise any impact on cost and introducing unexpected delays.

Although the role for HCV RNA point‐of‐care is becoming clearer in aiding diagnosis of viraemic individuals, there have been little data to support its confirming of post‐treatment response. As part of their Xpert^®^ HCV VL Fingerstick assay validation, Lamoury and colleagues reported a sensitivity of 81.8% and a specificity of 100% for the Xpert^®^ HCV VL Fingerstick assay to detect HCV RNA in a small group of patients on DAA treatment (n = 16).^16^ Additional work in larger cohorts using HCV core antigen as a surrogate measure for viraemia to evaluate sustained virological response at 12 weeks post‐therapy have reported high levels of concordance with HCV RNA levels.^36^ In order to truly decentralise and simplify future efforts to improve linkage to HCV treatment in marginalised settings, it is clear that HCV RNA point‐of‐care will play an important role. Therefore, it is vital that further evaluation of the utility, performance and cost‐effectiveness of HCV RNA point‐of‐care, such as Xpert^®^ HCV VL Fingerstick assay, is conducted to confirm HCV treatment response in resource‐limited settings.

It is also important to appreciate the role of HCV point‐of‐care diagnostic technology to enhance the linkage‐to‐care. There are emerging examples of the Xpert^®^ HCV VL assay improving screening and linkage‐to‐care from a community programme in Egypt and marginalised PWID screening in Australia and, reporting treatment uptake of 93% and 44% respectively.^37^, ^38^ A unique feature of the Xpert^®^ HCV VL Fingerstick assay is a rapid turn‐around time (60 minutes) making a same visit HCV RNA diagnosis a real possibility, which could potentially further improve linkage to treatment.

Despite our study providing further empirical evidence supporting the utility of the Xpert^®^ HCV VL Fingerstick assay to reliably diagnose viraemic HCV in resource‐limited settings, there are some notable limitations. Firstly, although this is a sizeable evaluation of Xpert^®^ HCV VL Fingerstick assay performance, it was conducted in a hospital environment and further evaluation in different challenging settings (such as rural areas) is still warranted. Secondly, the Xpert^®^ HCV VL assay was used as the gold standard as there was no local access to conventional laboratory‐based NAT testing. However, it is unlikely that this would have introduced any significant discrepancy as this assay is WHO pre‐qualified and has demonstrated excellent performance in previous validation studies.^25^, ^26^ Thirdly, as local genotyping was not available, only those with historic genotypes were reported in this study. However, though it is relevant from an epidemiological perspective to determine genotype, its clinical relevance has been minimised by the availability of pangenotypic DAAs, as proposed in a recent algorithm for screening in LMIC settings.^39^ Finally, our conclusions are limited to PWID engaged in harm reduction and determining whether Xpert^®^ HCV VL Fingerstick assay testing is reproducible and feasible in marginalised rural settings is important. Cepheid has recently launched a portable module (Cepheid Edge), which could serve to address this in the future.^40^

Until recently, access to HCV NAT confirmation was considered a major obstacle to improving access to care in resource‐limited settings. We provide evidence here that the Xpert^®^ HCV VL Fingerstick assay is a technically feasible, cost‐amenable and ultra‐sensitive assay to simplify HCV NAT diagnosis in resource‐limited settings and paves the way to facilitate the much‐needed HCV treatment intervention in our challenging PWID setting in sub‐Saharan Africa.

## CONFLICT OF INTEREST

The authors do not have any disclosures to report.
